# Research progress on morphology and mechanism of programmed cell death

**DOI:** 10.1038/s41419-024-06712-8

**Published:** 2024-05-10

**Authors:** Yao Chen, Xiaohua Li, Minfeng Yang, Song-Bai Liu

**Affiliations:** 1 Suzhou Key Laboratory of Medical Biotechnology, Suzhou Vocational Health College, Suzhou, China; 2 Department of Thyroid and Breast Surgery, Wuzhong People’s Hospital of Suzhou City, Suzhou, China; 3 School of Public Health, Nantong University, Nantong, 226019 China; 4 Department of Health Technology and Informatics, The Hong Kong Polytechnic University, Kowloon Hong Kong SAR, China; 5 State Key Laboratory of Radiation Medicine and Protection, Soochow University, Suzhou, 215123 China

**Keywords:** Cell death, Cell signalling

## Abstract

Programmed cell death (PCD) is a basic process of life that is closely related to the growth, development, aging and disease of organisms and is one of the hotspots of life science research today. PCD is a kind of genetic control, autonomous and orderly important cell death that involves the activation, expression, and regulation of a series of genes. In recent years, with the deepening of research in this field, new mechanisms of multiple PCD pathways have been revealed. This article reviews and summarizes the multiple PCD pathways that have been discovered, analyses and compares the morphological characteristics and biomarkers of different types of PCD, and briefly discusses the role of various types of PCD in the diagnosis and treatment of different diseases, especially malignant tumors.

## Facts

1. Different types of PCD have unique signaling pathways and specific morphological characteristics.
2. Clarifying the specific biomarkers and regulatory mechanisms of various PCD pathways and their relationships with specific diseases is vital for further targeted treatment.
3. Further studies on the molecular mechanism and mutual cross-links of various types of PCD and the identification of specific drug targets are the main directions in the future.

## Open questions

1. How many forms of PCD exist, and how can they be classified more scientifically?
2. Which PCD pathways are preferentially activated, and how can different PCD pathways be coordinated for the treatment of specific diseases?
3. How can the potential off-target effects of PCD induction be effectively prevented and controlled for disease therapy?

## Introduction

Cell death is a common biological phenomenon that plays an important role in the growth and development of the body and is related to the occurrence and development of a variety of diseases. Cell death includes accidental cell death (ACD) and programmed cell death (PCD). ACD is a kind of passive catastrophic cell death caused by extreme physical (such as high pressure, high temperature, high osmotic pressure, etc.), chemical, or mechanical damage, in which necrosis is the main type of ACD. Necrosis in ACD is an unregulated, passive form of cell death characterized by cell swelling, membrane rupture, organelle collapse, and the release of cell contents, often leading to an inflammatory response in the body. PCD is an active form of cell death that occurs under physiological conditions controlled by genes, and it is an important regulatory mechanism for the body to stabilize the internal environment and balance the number of cells in the physiological and pathological environment. It depends on a special molecular mechanism and can be regulated (i.e., delayed or accelerated) through drug or gene intervention. It is worth noting that some forms of PCD discovered in recent years, such as necroptosis and pyroptosis, also show characteristics similar to those of necrosis in ACD, including membrane rupture and inflammatory response; however, unlike necrosis in ACD, these processes are regulated and can be affected by inhibitors, activators, protein expression levels, etc.

PCDs present visible morphological changes, Schweichel et al. classified PCD into three distinct morphological types based on morphology combined with the mechanism of cell death [1, 2]: (1) type I cell death or apoptosis; (2) type II cell death or autophagy; and (3) type III cell death or necrosis. Moreover, each type of PCD involves a specific molecular signaling pathway and regulatory system [3]. These regulated forms of cell death are intrinsically related to human embryonic development, homeostasis maintenance, and disease pathology, indicating broad prospects for clinical application in this field. With the continuous development of this field, new signaling pathways that regulate PCD are still being described.

**Fig. 1:**
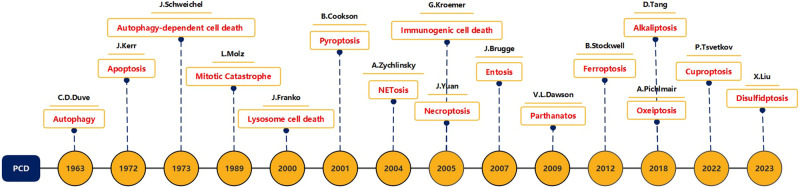
Timeline of PCD discovery. The discoverer and time of various types of PCD.

In this article, we reviewed and analyzed the definitions, morphological characteristics, molecular mechanisms, and biomarkers of the main PCD types identified to date, which include apoptosis, necroptosis, pyroptosis, ferroptosis, autophagy-dependent cell death, mitotic catastrophe, immunogenic cell death, entosis, parthanatos, ferroptosis, disulfidptosis, NETosis, lysosome-dependent cell death, alkaliptosis, and oxeiptosis (Fig. 1), and briefly described the role and prospects of these major types of PCD in related diseases.

## Morphological classification of the PCDs

In 1990, Clarke et al. supplemented Schweichel and Merker’s classification of cell death by dividing PCD into three types [4]. Type I refers to apoptosis (condensation, fragmentation, or phagocytosis). It is characterized by nuclear condensation and pyknosis, cell membrane coiling and blistering, cell size reduction, ribosome dissociation from polysomes and rough endoplasmic reticulum, and elimination of dead cells by heterophagolysosomes (heterophagy, engulfed by phagocytes after death). Type II is autophagic degeneration. It is characterized by the production of inwards bubbles in the cell membrane (endocytosis), the production of abundant autophagic vacuoles in the cytoplasm, the general expansion of the endoplasmic reticulum, mitochondria and Golgi apparatus, less obvious nuclear pyknosis than type I, and the elimination of dead cells by autolysosomes (autophagy). Type III is nonlysosomal vesicular degradation characterized by shrinkage and rounding or fragmentation of the cell membrane, edema, and dissolution or fragmentation of the nucleus, expansion of the endoplasmic reticulum, mitochondria, and Golgi apparatus, absence of early karyknosis, and dead cells that are not eliminated by lysosomes (cell dissolution in situ). Although this morphological classification is still widely used, it is mainly based on the phenotypes of the three pathways of apoptosis, autophagy, and necrosis, and these three types of characteristics are not fully representative of the other pathways in the currently known PCD type. Therefore, we optimized and supplemented the classical morphological classification, classified the known PCD pathways according to the characteristic morphology of the dead cells, how they were eliminated, whether they were accompanied by an inflammatory reaction, etc., and summarized the unique characteristics of various PCD pathways, thus providing a theoretical basis for the identification of various PCDs (Table 1).

**Table 1:** Morphological types of PCD and their specific characteristics.

Morphological types	PCD pathway	Specific morphological characteristics
Type I cell death (Apoptosis-like PCD) Cell shrinkage, Chromatin condensation, Eliminated by heterophagolysosome, Without inflammation.	Apoptosis	Cell contraction, Chromatin condensation, Small DNA fragments, Apoptotic body.
	Mitotic catastrophe	Chromatin Condensation, Multiple micronucleated giant cells. (most commonly induces Apoptosis)
	Immunogenic cell death	Morphological characteristics like Apoptosis.
	Oxeiptosis	Morphological characteristics like Apoptosis.
Type II cell death (Autophagy-like PCD) Cytoplasmic vacuolization, Formation of autophagic vesicles, Eliminated by autolysosomes.	Autophagy-dependent cell death	Inwards bubble of membrane, Autophagosomes form and aggregate.
	Entosis	Cell-in-cell structure.
	Lysosome-dependent cell death	Lysosome rupture.
Type III cell death (Necrosis-like PCD) Cells and organelles edema, Cell membrane destruction, Chromatin does not condense, Without the participation of lysosomes, With inflammation.	Necroptosis	Cells and organelles edema, Cell membrane disruption, Chromatin does not condense, Cell content release with inflammatory response.
	Pyroptosis	Morphological characteristics like Necroptosis.
	Ferroptosis	Mitochondrial atrophy, Mitochondrial membrane thickens, Mitochondrial membrane disruption, Mitochondrial ridge reduction.
	Parthanatos	Cell contraction, Chromatin Condensation, Large DNA fragments, Cell membrane disruption.
	Cuproptosis	Mitochondrial shrinkage, Mitochondrial membrane disruption.
	Disulfidptosis	Cell shrinkage, F-actin contraction and detachment from the plasma membrane.
	NETosis	Cell membrane rupture, Release network structure.
	Alkaliptosis	Morphological characteristics like Necroptosis.

## Molecular mechanisms of the PCDs

### Apoptosis

**Fig. 2:**
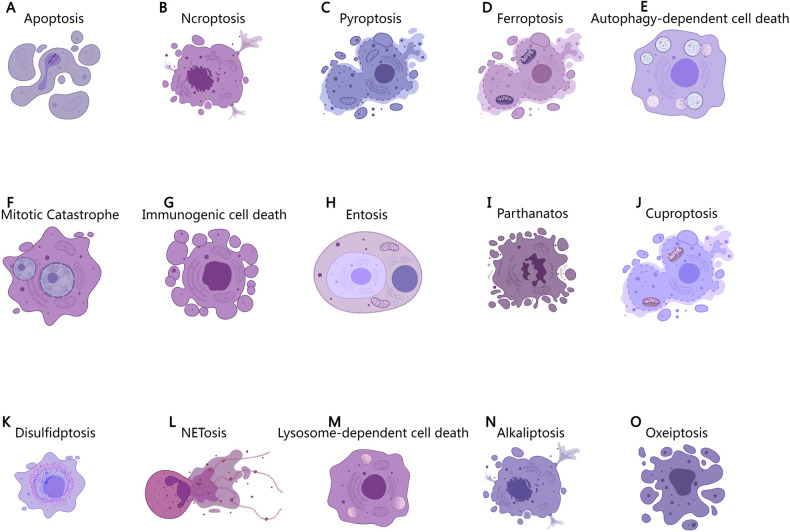
Morphological characteristics of various types of PCD. **A** Apoptosis: cell contraction, chromatin condensation, small DNA fragments, and apoptotic body; **B** necroptosis: cell membrane disruption, cell and organelle edema, chromatin does not condense, cell content release with inflammatory response; **C** pyroptosis: morphological characteristics like necroptosis; **D** ferroptosis: mitochondrial atrophy, mitochondrial membrane thickening, mitochondrial membrane disruption, mitochondrial ridge reduction; **E** autophagy-dependent cell death: cell membrane disruption, cytoplasmic vacuolation, autophagosomes form and aggregate; **F** mitotic catastrophe: chromatin condensation, multiple micronucleated giant cells; **G** immunogenic cell death: morphological characteristics like apoptosis; **H** entosis: cell-in-cell structure; **I** parthanatos: cell contraction, chromatin condensation, large DNA fragments, cell membrane disruption; **J** cuproptosis: mitochondrial shrinkage, mitochondrial membrane disruption; **K** disulfidptosis: cell shrinkage, F-actin contraction and detachment from the plasma membrane; **L** NETosis: cell membrane rupture, release network structure; **M** lysosome-dependent cell death: lysosome rupture; **N** alkaliptosis: morphological characteristics like necroptosis; **O** oxeiptosis: morphological characteristics like apoptosis.

Apoptosis is a programmed and active death process that occurs in cells under the control of specific genes or pathways [5]. The morphological characteristics of apoptosis are as follows: disappearance of cell junctions, reduction in volume, condensation of nuclear chromatin, nuclear lysis, cytoplasmic contraction, dilation of the endoplasmic reticulum and cell membrane blistering, and finally, division into apoptotic bodies by the cell membrane (Fig. 2A). Apoptotic bodies contain a variety of different fragments of organelles and chromatin with intact structures. The morphology of the mitochondria remained unchanged. Apoptosis is accompanied by the entire process of individual growth and development, showing the precise control of the type and number of cells.

**Fig. 3:**
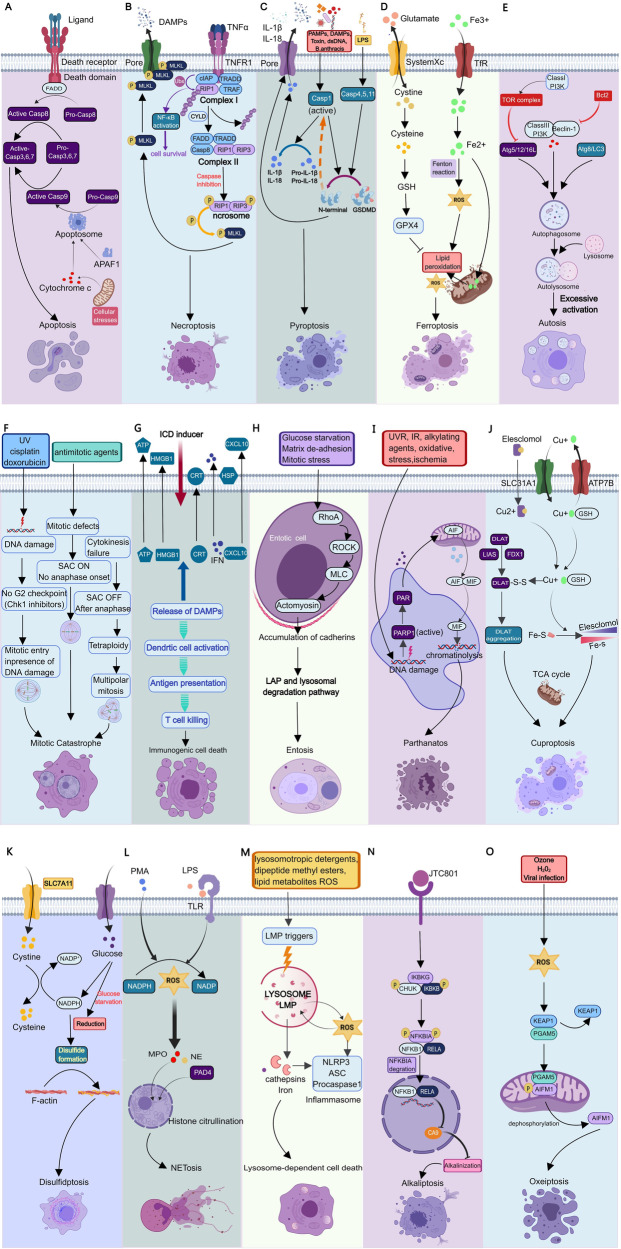
Signaling pathways of various types of PCD. **A** Apoptosis; **B** Necroptosis; **C** Pyroptosis; **D** Ferroptosis; **E** Autophagy-dependent cell death **F** Mitotic catastrophe; **G** Immunogenic cell death; **H** Entosis; **I** Parthanatos; **J** Cuproptosis **K** Disulfidptosis; **L** NETosis; **M** Lysosome-dependent cell death; **N** Alkaliptosis; **O** Oxeiptosis.

The typical process of apoptosis involves a series of hydrolysis, caspase activation, and signal transduction processes; thus, this process is also called the caspase-dependent apoptosis pathway. Due to the different sources of apoptosis signals, classical apoptosis is divided into intrinsic apoptosis (mitochondrial apoptosis pathway) and extrinsic apoptosis (death receptor pathway) [6]. Intrinsic apoptosis is a form of PCD that initiates apoptosis due to disturbance of the intracellular microenvironment. Intrinsic apoptosis can be induced by growth factor deficiency, DNA damage, endoplasmic reticulum pressure, excess reactive oxygen species (ROS), replication pressure, microbundle changes, and mitotic defects [7]. The most important step in intrinsic apoptosis is mitochondrial outer membrane permeabilization (MOMP), which is mainly controlled by the BCL2 protein family. Among the inner and outer membranes of mitochondria, there are two main proapoptotic factors: cytochrome C (Cyt-C) and apoptosis-inducing factor (AIF). Apoptosis signals cause Cyt-C to be released from mitochondria to the cytoplasm, after which it binds to apoptotic protease activating factor 1 (APAF1), initiates a series of cascade reactions, and finally activates deoxyribonucleases and hydrolyzes nucleic acid and cytoskeleton proteins, leading to apoptosis; thus, the intrinsic pathway is also called the mitochondrial apoptosis pathway. Extrinsic apoptosis is a form of PCD caused by disturbance of the extracellular microenvironment. The extrinsic pathway induces apoptosis by activating specific death receptors on the cell surface through extrinsic death signals; thus, this pathway is also called the death receptor pathway [8]. At present, eight types of death receptors have been found, among which Fas (also known as CD95 or Apo-1) and tumor necrosis factor receptor (TNFR) are the most important. These death receptors contain a death domain of approximately 80 amino acids, which is necessary to mediate apoptosis [9, 10]. The apoptotic process of the death receptor pathway needs to be mediated by caspase8 activation [11]. Stimulation of the Fas receptor leads to its binding to the Fas ligand and Fas-associated death domain (FADD). FADD binds to pro-caspase8 to form a death-inducing signaling complex (DISC). Then, the DISC recruits pro-caspase8 to induce its activation through its own splicing, initiating the downstream caspase pathway and leading to apoptosis [12]. Both the intrinsic and extrinsic apoptosis pathways eventually undergo a cascade amplification process involving the irreversible limited hydrolysis of substrates mediated by caspase family members, which act on substrates and lead to apoptosis [13] (Fig. 3A). Caspases can be divided into two functional categories: inflammatory caspases, which include mainly caspase-1/4/5/11, which are involved in the processing of cytokines and the regulation of inflammation. The other type is proapoptotic caspases, which mainly include caspase-2/3/6/7/8/9/10, which are cascades involved in the process of apoptosis. The morphological characteristics of apoptosis are caused by proapoptotic caspases cutting intracellular proteins and inducing DNA cutting [14]. Caspases related to apoptosis can be divided into initial caspases (caspase 2/8/9/10) and effector caspases (caspase-3/6/7). These kinds of apoptosis regulatory factors need to be activated by the cleavage of pro-caspase. Downstream of the apoptosis pathway, its key biomarker is cleaved and activated caspase 3, making the cell irreversible to apoptosis. In addition, phosphatidylserine (PS), which is normally localized in the inner leaflet of the phospholipid bilayer of the cell membrane, is flipped to the outer leaflet when apoptosis occurs. Therefore, caspase3/8 cleavage and PS evagination can be used as the gold standards for detecting apoptosis. The study of apoptosis can not only be used for the early detection of cancer and improve the survival rate of cancer patients but can also be treated accurately and quickly by inducing tumor cell apoptosis.

### Necroptosis

Necroptosis is a form of PCD that is controlled by a unique caspase-independent signaling pathway and has necrosis-like morphological characteristics [15]. Compared with necrosis in ACD, necroptosis involves the same subcellular changes, such as a sharp increase in intracellular peroxide, high phosphorylation of the mitochondrial membrane, increased membrane permeability, cell swelling, and cell membrane destruction (Fig. 2B). However, necroptosis is regulated by a variety of genes and is a regular mode of cell death. In contrast to apoptosis, necroptosis does not involve the formation of apoptotic bodies, and chromatin does not agglomerate.

Necroptosis can be triggered by Toll-like receptor activation, ROS accumulation in mitochondria, tumor necrosis factor-α (TNF-α), and viral infection. When stimulated, caspase-inhibited cells may undergo necroptosis rather than apoptosis [16]. For example, TNF-α induced necroptosis in mouse fibroblast cells treated with the pan-caspase inhibitor Z-VAD-fmk, and it also caused necroptosis in caspase-8-deficient leukemic Jurkat cells. Thus, necroptosis may be the mechanism by which cells die when apoptosis fails to initiate normally [17]. Different cells undergo apoptosis or necroptosis according to their environment and degree of activation. In the signaling pathway, TNF-α binds and activates TNFR1 to induce necroptosis. After TNF-α binds TNFR1, TNFR1 recruits a series of proteins to form different complexes on the cytoplasmic side. Among them, complex I include TNFR-associated death domain (TRADD), receptor-interacting protein kinase 1 (RIPK1), TNFR-associated factor 2 (TRAF2), TRAF5, cellular inhibitor of apoptosis protein 1 (cIAP1), cIAP2, and the ubiquitinase complex. At this point, if RIPK1 is polyubiquitinated, it will further form a complex to activate the signaling pathway mediated by nuclear factor-κB (NF-κB) and mitogen-activated protein kinase (MAPK) and subsequently inhibit cell death [18]. TNFR1 dissociates from complex I, and RIPK1 deubiquitinates and forms complex II with RIPK3, TRADD, and Fas-associated protein via a death domain (FADD) and caspase-8. In this complex, if caspase-8 inactivates RIPK1 and RIPK3, the cell will die by apoptosis; if caspase-8 is inhibited, RIPK1 and RIPK3 bind to each other through their respective RHIM domains to form a necroptosis complex. In this case, RIPK3 autophosphorylates, recruits, and phosphorylates MLKL, and then initiates necroptosis [19, 20]. MLKL can serve as a platform for the recruitment of Ca^2+^ and Na^+^ in the plasma membrane and can also play a role in the formation of pore complexes in the membrane [21, 22]. Therefore, the activation of MLKL by RIPK3 is a key regulatory pathway of necroptosis, and the phosphorylation of MLKL has become a biomarker of necroptosis [23] (Fig. 3B). The occurrence of necroptosis can be marked by detecting the phosphorylation of key necroptosis molecular markers, such as RIPK1, RIPK3 and MLKL, while necrostatin-1, a specific inhibitor of RIPK1, can block the occurrence of necroptosis. Necroptosis is involved in a variety of pathological processes in the body, such as bacterial and viral infections or inflammatory lesions caused by sterile lesions such as atherosclerosis. In addition, necroptosis is also considered a possible barrier against tumor formation.

### Pyroptosis

Pyroptosis is a form of PCD that is related to the innate immune response (such as pathogen invasion) and depends on Gasdermin family proteins to form plasma membrane pores [24, 25]. This process is often but not always completed by the activation of inflammatory caspases, resulting in necrosis-like morphological characteristics similar to those of necrosis in ACD, including nuclear fragmentation and dissolution; increased cell membrane permeability; swelling and lysis; and the release of cellular contents, which cause a local inflammatory response (Fig. 2C).

The molecular mechanisms of pyroptosis include classical and nonclassical pyroptosis pathways. Classical pyroptosis mainly depends on the activation of Caspase-1. When pathogens invade host cells, caspase-1 can be activated by binding to inflammatory bodies (pyroptosomes), which are a variety of protein signaling complexes, and their central scaffolds include the inflammatory bodies NLRP1, NLRP3, NAIP-NLRC4, AIM2 and pyrin [26]. After caspase-1 activation, a heterodimer with enzyme activity is formed, which specifically cleaves GSDMD in the Gasdermin domain protein family to release the N-terminal domain, inserts the cell membrane lipid bilayer, and induces oligomerization in the membrane to form pores [27], resulting in the destruction of cell membrane osmotic pressure, cell swelling and rupture, content release and inflammation, resulting in pyroptosis. Moreover, the activation of caspase-1 can also promote the activation and secretion of the inflammatory cytokines IL-18 and IL-1β to recruit more immune cells to the infected site and expand the inflammatory response. Nonclassical pyroptosis is premised on the activation of caspase-4,5,11. Caspases 4, 5, and 11 can be activated by direct binding to bacterial lipopolysaccharide (LPS), and activated caspases 4, 5, and 11 can also lead to pyroptosis by cleaving GSDMD, so caspase cleavage of the GSDMD substrate is a key execution event of pyroptosis [28]. In addition, upon specific binding of LPS to caspase-11, activated caspase-11 can also activate the Pannexin-1 transmembrane channel, causing the outflow of the intracellular danger signal molecule ATP and the opening of nonselective positive ion channels. Then, the outflow of intracellular K^+^ and the influx of extracellular Na^+^ and Ca^2+^ will damage the integrity of the cell membrane and release the inflammatory contents in the cells, which will trigger the inflammatory response [29]. Other studies have shown that caspase-1 is also involved in the nonclassical pyroptosis pathway mediated by Caspase-11, and the activation of caspase-1 can release the proinflammatory factors IL-1β and IL-18 [30] (Fig. 3C). The specific biomarker of pyroptosis is the cleavage of Gasdermin family proteins, so pyroptosis can be indicated by detecting the N-terminal domain released after the cleavage of Gasdermin proteins. Pyroptosis is an important immune response of the body that is closely related to infectious diseases, cardiovascular diseases, nervous system diseases, and tumors.

### Ferroptosis

Ferroptosis is a type of PCD caused by iron-dependent oxidative damage. Ferroptosis is characterized by disordered intracellular iron ion flow and significant increases in ROS and lipid peroxide levels without the need for caspases [31–35]. Ferroptosis is completely different from other forms of death in morphology, biochemistry, and genetics. The formation of apoptotic bodies, DNA fragmentation, and activation of the Caspase family are not observed in ferroptosis, and these effects cannot be reversed by Caspase inhibitors. The morphological characteristics of ferroptosis mainly include increased mitochondrial membrane density, a significant reduction in the number of mitochondrial ridges, membrane rupture, and overall mitochondrial atrophy; the nuclei were intact and normal in size, and no condensation of chromatin was observed; the cell membrane did not bleb, but the membrane density increased; and both the structure of the membrane phospholipid bilayer and the fluidity of the cell membrane changed [36] (Fig. 2D).

A variety of substances and external conditions can trigger ferroptosis, and the main stimulus signals are related to phospholipids. Ferroptosis is caused by the accumulation of ROS on membrane lipids due to the lack of the membrane lipid repair enzyme glutathione peroxidase (GPX4), and this accumulation requires iron ions. During ferroptosis, a large amount of free Fe^2+^ accumulates in the cell. Free Fe^2+^ is highly oxidized and prone to the Fenton reaction with H_2_O_2_, which produces hydroxyl radicals that can cause oxidative damage to DNA, proteins, and membrane lipids, promoting lipid peroxidation, damaging the cell membrane, and leading to cell death [37]. Lipid peroxidation refers to the loss of hydrogen atoms in lipids under the action of free radicals or lipid peroxidases, which leads to the oxidation, breakage, and shortening of lipid carbon chains and the production of lipid free radicals, lipid hydroperoxide (L-OOH), reactive aldehydes (malondialdehyde, 4-hydroxynonenal) and other cytotoxic substances, resulting in cell damage from lipid oxidative degradation [38]. GSH is an important antioxidant in the human body. It not only reduces H_2_O_2_ to H_2_O, a scavenger of free radicals, and maintains the balance of intracellular free radical content but also participates in the reduction of L-OOH as a cofactor of GPX4, repairing L-OOH in biofilms and preventing ferroptosis. The depletion of GSH leads to the inactivation of GPX4 and increases the lipid peroxidation reaction occurring on the inner side of the cell membrane phospholipids, which leads to membrane breakage, cell disintegration, and death. Thus, GSH depletion and inhibition of GPX4 enzyme activity are necessary for cells to undergo ferroptosis (Fig. 3D). Some substances have been found to be effective in inhibiting or promoting ferroptosis. Ferroptosis activators can be roughly divided into three categories according to their targets. The first class comprises System Xc inhibitors, such as erastin and sorafenib. Erastin decreases the uptake of cysteine by cells by inhibiting System Xc, leading to the depletion of GSH, the substrate of GPX4, and further reducing GPX4 activity, leading to the accumulation of ROS and ferroptosis. The second class includes GPX4 inhibitors, such as RSL3 and FIN56. RSL3 can bind to GPX4 and inhibit its protein activity, leading to the accumulation of toxic L-OOH and triggering ferroptosis [39]. The third class includes GSH-depleting agents, such as cysteinase, BSO, and cisplatin. Cysteinase can directly degrade cysteine and block the synthesis of GSH [40]. In addition, some activators, such as ferric citrate, can trigger ferroptosis by inducing lipid peroxidation or increasing intracellular free iron ion levels. Most ferroptosis inhibitors are iron chelators or antioxidants, such as deferoxamine mesylate (DFO) and ferrostatin, which inhibit the production of lipid ROS by scavenging free iron ions, hydroxyl radicals, or lipid free radicals, thus effectively reducing or eliminating the damage caused by ferroptosis. The main causes of ferroptosis are an imbalance in cell metabolism and the accumulation of ROS, which can be confirmed by detecting the degree of ROS accumulation and ROS products in cells. Furthermore, there is some more direct evidence. For example, ferroptosis can be inhibited by several known ferroptosis inhibitors (iron chelators, antioxidants, etc.), and relatively specific morphological phenomena, such as mitochondrial atrophy, reduction or even disappearance of mitochondrial ridges, and increased membrane density, can be observed under an electron microscope to prove the occurrence of ferroptosis. In recent years, studies have shown that ferroptosis is associated with many diseases, such as Parkinson’s syndrome, breast cancer, and pancreatic cancer, among which malignant tumors are most closely related. Some tumor cells are quite sensitive to ferroptosis. For example, dihydroartemisinin can induce ferroptosis in squamous cell carcinoma of the head and neck, thereby inhibiting tumor growth [41]. Therefore, ferroptosis is expected to become a new direction for disease treatment.

### Autophagy-dependent cell death

Autophagy-dependent cell death (ADCD) is a kind of PCD that must be driven by the molecular mechanism of autophagy in the process of cell death [1, 42], and its morphological feature is the observation of double-membrane phagocytic components containing vesicles, namely, autophagosomes; the expansion and fragmentation of the endoplasmic reticulum, mitochondria and other organelles; and the slight agglutination of chromatin [43, 44] (Fig. 2E).

Autophagy is a highly conserved catabolic mechanism that relies on lysosomes to degrade aging components in cells. It is often triggered by conditions such as nutrient deprivation, pathogen infection, hypoxia, or endoplasmic reticulum stress. Cells use a double-layer membrane structure to enclose pathogens, cytoplasmic proteins, or organelles to form autophagosomes or vesicles. Then, these substrates fuse with lysosomes and are degraded, thereby removing excessive or abnormal proteins, organelles, or pathogenic microorganisms. This mechanism is conducive to the maintenance of cell homeostasis and the renewal of organelles. However, excessive autophagy can lead to cell death, that is, ADCD, indicating the existence of a threshold effect of autophagy. The regulatory mechanism of autophagy is very complex and involves multiple regulatory pathways and regulators. According to current studies, autophagy is regulated mainly by the phosphatidylinositol 3-phosphate kinase-mammalian target of rapamycin (PI3K-mTOR) signal transduction pathway upstream of autophagy-associated genes (ATG) and the Beclin1 complex [45, 46]. Type I PI3K induces the generation of phosphatidylinositol 3,4,5-triphosphate (PIP3) in response to a stimulus signal. Under the action of 3-phosphatidylinositol-dependent kinase 1 (PDK1), protein kinase B (AKT) binds to PIP3 and is activated to form phosphorylated AKT. It further acts on the downstream target protein mTOR, thereby negatively regulating autophagy [38]. The mTOR pathway is considered to be the “gatekeeper” and “monitor” of autophagy and has an inhibitory effect on autophagy [47]. ATG can also form complexes with other components to regulate autophagy. Beclin1, the first autophagy-related gene discovered, regulates the formation of autophagosomes by binding to type III PI3K to form complexes, thereby promoting the occurrence of ADCD [48]. The combination of Beclin1 and Bcl2 inhibited autophagy. Two ubiquitin-like pathways, the Atg5-Atg12-Atg16L complex, and the Atg8/LC3 system, play crucial roles in autophagosome formation (Fig. 3E). Therefore, LC3 lipidation and an increase in the LC3-II/LC3-I ratio are considered biomarkers of autophagy. Autosis is a subtype of ADCD that relies on the plasma membrane Na^+^/K^+^-ATPase [43]. It can be induced by nutrient deprivation or by Tat-Beclin1 and inhibited by blocking upstream Na^+^/K^+^-ATPase, which is a plasma pump linking ion homeostasis and ER stress [43]. ADCD may play an important role in neuronal cell death induced by neurotoxicity or hypoxia-ischemia.

### Mitotic catastrophe

Mitotic catastrophe (MC) is a regulated tumor suppressor mechanism in which the mitotic process of cells is dysregulated due to DNA damage or other causes, resulting in cell death accompanied by tetraploid or polyploid formation [49]. It was first identified in a heat-sensitive yeast mutant strain that dies as a result of abnormal chromosome segregation during cell division [50, 51]. Its main morphological features are nuclear micronization and polykeratosis (cell size becomes larger, forming giant cells containing two or more nuclei and partially condensed chromatin) [52] (Fig. 2F).

Current studies have shown that DNA damage, mitotic defects, and cytokinesis failure are the main causes of MC, which are regulated by a variety of molecules and closely related to cell cycle checkpoints and cell cycle-related kinase abnormalities. The induction of MC in cells can constitute a new target for cancer therapy. DNA damage can be caused by internal factors (such as replication errors or cell-generated ROS) or external environmental factors (such as radiation and platinum compounds), thereby disrupting the integrity and stability of the cellular genome. When the cell undergoes DNA damage and the G2 checkpoint is defective or damaged in the cell cycle, the cells with DNA damage enter the M phase of mitosis prematurely through the G2 checkpoint, resulting in chromosome segregation error and subsequent MC [53]. Therefore, an abolished or defective G2 checkpoint is essential for DNA damage-induced MC. In addition, during mitosis, the precise segregation of sister chromosomes is controlled by the spindle assembly checkpoint (SAC), also known as the mitotic checkpoint [54]. The SAC can prevent cells from entering anaphase during mitosis before the bilateral kinetochore of all sister chromosomes forms proper attachments to their respective bipolar spindle microtubules to prevent errors during chromosome segregation. The mitotic checkpoint signaling pathway is activated when spindle microtubules do not connect or misconnect kinetosomes on both sides of chromosomes due to reasons such as a lack of mitotic organs. After the initiation of SAC, it can effectively inhibit the activity of the anaphase-promoting complex (APC) and prevent the continuation of mitosis [53]. However, long-term activation of the SAC can lead to mitotic arrest, resulting in mitotic defects in cells and, thus, in MCs [55]. Therefore, when the mitotic checkpoint signal is destroyed or activated for a long time, the cell can initiate anaphase or stay in metaphase before the chromosome kinetosomes have all established the correct connection with the spindle microtubule, resulting in mitotic defects, chromosome missegregation, and then aneuploidy, which further leads to the occurrence of MC. Defects in cyclins or related kinases can also lead to the inhibition of cytokinesis, resulting in polyploidy and multipolarized mitosis at anaphase of mitosis, resulting in genomic instability and stimulating MCs in the next cell cycle [53] (Fig. 3F). Related kinases include mitotic kinases such as Aurora kinases, monopolar spindle 1 (MPS1), and polo-like kinases (Plks), which play key roles in proper chromosome segregation. The exact molecular mechanism by which mitotic alterations are sensed and trigger the MC cascade is unknown, but p53 may be involved [56]. Extensive experimental evidence suggests that MC is facilitated by a signal transduction cascade dependent on caspase-2 activation, often (but not always) triggering intrinsic apoptosis regulated by the BCL2 protein family [57]. However, it has also been found that in certain cases where p53 is lacking, mitotic defects appear to drive a necrotic variant of PCD independent of caspase-2 signaling [58]. In addition, although apoptosis usually occurs when cells are in an abnormal mitotic state for a long time, there are rare cases in which MC does not lead to cell death but eventually results in senescence, a type of irreversible cell cycle arrest [59]. Thus, MCs do not always cause PCD (and can also drive cellular senescence), and the Committee on Cell Death (NCCD) recommends the use of the term mitotic death to denote PCD driven by mitotic mutations (most commonly intrinsic apoptosis) [25]. MC is biologically characterized by low expression or absence of proteins associated with G2/M phase checkpoints and mitotic spindle assembly. The detection methods mainly include optical microscopy, laser confocal microscopy, or electron microscopy to detect cells containing two or more nuclei, and flow cytometry to detect G2/M block and polyploidy. In recent years, inducing tumor cells to cross the cell cycle checkpoint with a large amount of DNA damage or errors, leading to MC and death of tumor cells, has made an important attempt at clinical treatment and targeted drug development for cancer.

### Immunogenic cell death

Immunogenic cell death (ICD) is a specific variant of PCD that is driven by stress and can induce adaptive immune responses against dead cell antigens [60, 61]. The morphological features of ICD were similar to those of apoptosis (Fig. 2G).

ICD can be caused by a variety of different stressors, including but not limited to (1) intracellular pathogens; (2) traditional chemotherapeutic drugs such as anthracyclines, DNA damage agents, and proteasome inhibitors; (3) targeted anticancer drugs such as crizotinib (a tyrosine kinase inhibitor), cetuximab (an epidermal growth factor-specific monoclonal antibody), and poly-ADP ribose polymerase (PARP) inhibitors; (4) A variety of physical therapies, including radiotherapy, external photochemotherapy, photodynamic therapy, near-infrared immunotherapy, and nanopulse stimulation [62–65]. These stressors stimulate cells to produce a series of signaling molecules called damage-associated molecular patterns (DAMPs). DAMPs released during ICD can bind to pattern recognition receptors (PRRs) on the surface of dendritic cells (DCs) and initiate a series of cytological responses that ultimately activate innate and adaptive immune responses [66]. At this point, when the target cells exhibit sufficient antigenicity, their death is executed by cytotoxic T lymphocytes (CTLs), which induce target cell apoptosis through the perforin/granzyme pathway and the death receptor pathway and trigger an adaptive immune response involving immune memory (Fig. 3G). To date, six DAMPs have been linked to the immunogenic mechanism of ICD: (1) calreticulin (CALR/CRT) [67], (2) adenosine triphosphate (ATP) [68], (3) high mobility group box protein B1 (HMGB1) [69], (4) type I interferon (IFN) [70], (5) cancer cell-derived nucleic acids [67, 71], and (6) annexin A1 (ANXA1) [72]. The DAMPs released under the above different induction conditions can be used as biomarkers for subsequent studies. ICD and its DAMPs provide a new therapeutic basis and means for tumor therapy, monitoring changes in tumor cell immunogenicity before and after chemotherapy, and combining chemotherapy and immunotherapy can improve the therapeutic effect on tumors. In the treatment of tumors, chemotherapy drugs or radiotherapy induce the death of tumor cells and upregulate the expression of certain immune signaling molecules, such as CALR, on the surface of target cells. These signaling molecules enhance the ability of DCs to recognize tumors and present antigens. After DCs are stimulated to mature, they activate tumor-specific CTLs to attack tumors and stimulate the release of interleukin (IL)-2, IL-4, and IFN-γ to obtain more ideal antitumour therapeutic effects.

### Entosis

Entosis, also known as entotic cell death, is a form of cellular cannibalism that occurs in healthy and malignant mammalian tissues and is characterized by one cell engulfing and killing another and the formation of a “cell-in-cell” structure (CIC) [73, 74] (Fig. 2H).

Cell adhesion and cytoskeletal rearrangement pathways play important roles in the control of entosis induction and occurrence. Entosis is activated to phagocytose and kill similar cells through LC3-associated phagocytosis (LAP)- and cathepsin B (CTSB)-mediated lysosomal degradation pathways. After detachment from the extracellular matrix (ECM), epithelial cells first adhere to adjacent epithelial cells through the adhesion proteins E-cadherin/cadherin-herin 1 (CDH1) and alpha-E-catenin. Subsequently, the bacterium will invade another cell through actomyosin complex formation, the lysosomes of the invading cell will encapsulate the invasive cell, and the invasive cell will die through the lysosomal pathway [75]. There are two key factors that regulate entosis. One is the adhesion of cadherin, so calcium blockers can prevent entosis; the first adhesion protein family molecule, PCDH7, which negatively regulates entotic CIC structure formation, has also been recently discovered [76]. Another key factor is the Rho-ROCK signaling pathway, in which the expression of actomyosin complexes in the cytoskeleton is dependent on RAS homolog gene family member A (RhoA), Rho-associated protein kinase 1 (ROCK1), ROCK2 and related diaphanous factor (DIAPH1), which accumulate local activity. Therefore, blocking the Rho-ROCK signaling pathway can prevent cell invasion, thereby preventing the occurrence of entosis [73] (Fig. 3H). Studies have shown that relative intracellular Rho GTPase activity is a key factor regulating the selective clearance behavior of entosis. Malignant tumor cells with low Rho GTPase activity easily deform and engulf normal cells or benign tumor cells with high Rho GTPase activity. Therefore, in the pathological context of tumors, entosis is thought to be a mechanism of cell competition to select the best tumor cell clones by internalizing and killing failing cells [77] and to promote tumor evolution by inducing genomic instability in external cells [78]. Moreover, its role in the physiological environment has gradually been revealed. Recent studies have revealed the mechanism of mitosis surveillance in patients with entosis. This mechanism selectively promotes the penetration of aneuploid daughter cells into adjacent cells to form CIC structures by activating the p53 signaling pathway, and CIC is subsequently cleared to maintain the genomic stability of epithelial cells [79].

### Parthanatos

Parthanatos, also known as poly-ADP ribose polymerase-1 (PARP-1)-dependent cell death, is a new form of PCD in which PARP-1 is overactivated due to DNA damage [80]. It should be noted that in addition to DNA damage, oxidative stress, hypoxia, hypoglycemia, or inflammatory signals may also trigger the production of parthanatos [80]. The morphological characteristics of parthanatos are cellular atrophy, chromatin concentration, and plasma membrane rupture. Parthanatos had some characteristics similar to apoptosis and necrosis-like morphology, but there were also obvious differences. Compared with apoptosis, parthanatos cannot form apoptotic bodies or small DNA fragments. Compared with necrosis in ACD, parthanatos is unable to induce cell and organelle swelling or cytolysis [81] (Fig. 2I).

Mechanistically, the release of apoptosis-inducing factor mitochondrion-associated 1 (AIFM1) from mitochondria-mediated by poly-ADP ribose (PAR) or calpain may be responsible for the effects of parthanatos. When PARP-1 is activated in large quantities, it can use coenzyme I (NAD) and ATP as substrates to catalyze the synthesis of PAR, and PAR induces the migration of AIFM1 in mitochondria (referring to the process of AIFM1 entering the nucleus from mitochondria), which further induces chromatin condensation and large DNA fragmentation to complete the transmission of death signals [82] (Fig. 3I). Some scholars also believe that the release of AIFM1 is dependent on calpain activation and that calpain I can promote the formation and release of mature AIF in a cell-free state [83]. Thus, parthanatos has distinct biochemical characteristics, such as rapid activation of PARP-1, early PAR accumulation, changes in mitochondrial permeability, AIFM1 migration from mitochondria to the nucleus, and intracellular NAD and ATP consumption. Parthanatos is involved in a variety of diseases of the human body and plays a key role in various nervous system diseases.

### Cuproptosis

Cuproptosis is a new form of PCD that depends on the accumulation of intracellular copper and is triggered by the direct combination of copper with the fatty acylated components of the tricarboxylic acid cycle (TCA) [84]. The morphological features of cuproptosis include mitochondrial shrinkage, cell membrane disruption, and endoplasmic reticulum and chromatin damage (Fig. 2J).

Excess copper promotes the aggregation and functional loss of lipoacylated proteins, triggers the loss of iron-sulfur cluster proteins, blocks the TCA cycle, and leads to cytotoxic stress and eventual death. The addition of Elesclomol alone did not affect cell growth, but once copper ions were added, cell growth was inhibited. Therefore, the induction of cell death by copper-binding molecules or copper ionophores mainly depends on the availability of copper. In addition, cuproptosis requires the involvement of mitochondrial respiration, and copper is not directly involved in the electron transport chain (ETC) but only plays a role in the TCA cycle. It was subsequently confirmed that the key regulatory gene of cuproptosis is FDX1, which is the upstream regulator of protein lipoacylation. FDX1 encodes a protein that is directly targeted by Elesclomol, which converts Cu^2+^ to more toxic Cu^+^ and catalyzes the lipoacylation of DLAT, DLST, and LIST in the pyruvate dehydrogenase complex. Cu^+^ further binds to the lipoacylation site of DLAT, resulting in the oligomerization of DLAT, thus resulting in copper toxicity (Fig. 3J). Loss of FDX1 leads to a complete loss of protein lipoacylation while also causing a significant decrease in cellular respiration and attenuating copper ionophore-induced cell death. The abundance of FDX1 and lipoacylated proteins is highly correlated with a variety of human tumors. Cell lines with high levels of lipoacylated proteins were shown to be more sensitive to cuproptosis. This finding suggests that copper ionophores may be a potential therapeutic approach for targeting cancer cells with such metabolic profiles. Such a mechanism could explain the pathology associated with inherited copper overload diseases and suggest novel approaches to exploit copper toxicity in the treatment of cancer.

### Disulfidptosis

Disulfidptosis is a form of PCD that differs from apoptosis, ferroptosis, etc., and has not been characterized previously. Disulfide stress causes rapid death caused by excessive intracellular cystine accumulation in glucose-starved SLC7A11-overexpressing cells [85].

Solute carrier family 7 member 11 (SLC7A11) is a cystine/glutamate antiporter that is mainly involved in the transport of amino acids on the plasma membrane. It is also an important pathway for cancer cell survival. Studies have shown that the process of SLC7A11-mediated reduction of ingested cystine to cysteine is highly dependent on the reduced nicotinamide adenine dinucleotide phosphate (NADPH) generated by the glucose-pentose phosphate pathway [86]. Therefore, during glucose deprivation, NADPH in SLC7A11-overexpressing cells is rapidly depleted, and cystine and other disulfides abnormally accumulate, which induces disulfide stress and rapid cell death. Further studies revealed that thiol oxidants promoted cell death in cells with high SLC7A11 expression under glucose starvation conditions and led to a sharp accumulation of intracellular disulfide molecules. However, reducing agents of disulfide stress, such as dithiothreitol (DTT), β-mercaptoethanol (2ME), and TCEP, can completely inhibit glucose starvation-induced cell death in SLC7A11-overexpressing cells [85]. Unlike other cell death mechanisms, disulfidptosis is associated with the actin cytoskeleton. In SLC7A11-overexpressing cells, the proteins with the most significant increase in disulfide bonds during glucose deprivation were enriched mainly in biological processes or pathways related to the actin cytoskeleton and cell adhesion, which can cause abnormal disulfide bonds of actin skeleton proteins in cells and lead to subsequent F-actin contraction [85] (Fig. 3K). Thus, glucose starvation in SLC7A11-overexpressing cells induces significant changes in cell morphology, including cell shrinkage, F-actin contraction, and detachment from the plasma membrane (Fig. 2K). In addition, glucose is the starting material of glycolysis and is transported by the glucose transporter (GLUT) family through the cell membrane, so the GLUT inhibitors KL-11743 and Bay-876 can effectively inhibit glucose uptake, similar to glucose starvation. Studies have confirmed that GLUT inhibitors can induce disulfur status and cell death in cancer cells with high SLC7A11 expression, and cancer cell disulfidptosis may be a key factor in the therapeutic effect of GLUT inhibitors in the treatment of tumors with high SLC7A11 expression [86].

### Other types of PCD

NETosis, a form of PCD driven by neutrophil extracellular trap (NET) particles, is also known as NETotic cell death [87]. NETosis was initially found to be related to the extrusion of fibrous webs containing chromatin and histone proteins in neutrophils, which was later confirmed to be the extracellular network DNA protein structure released by cells in response to infection or injury [88]. The morphological characteristics of NETosis include nuclear swelling, nuclear membrane and cytoplasmic granule membrane lysis, cytoplasmic membrane rupture, chromatin contact with cytoplasmic granules, and subsequent discharge to the outside of the cell to form a grid structure (Fig. 2L). In NETosis, ROS and activated protein-arginine deiminase 4 (PAD4) are produced through the activation of NADPH oxidase, leading to chromatin densification and myeloperoxidase (MPO) and neutrophil elastase (NE) entry into the nucleus, promoting the expansion of additional chromatin and nuclear membrane destruction. After chromatin is released into the cytosol, it is decorated by cytoplasmic and granular proteins, which eventually leads to plasma membrane rupture, resulting in NET release and neutrophil death (Fig. 3L). Among them, histone citrullination and ROS production are needed. Originally identified as a means of neutrophil defense against pathogens, NETosis also occurs in aseptic inflammation, promotes thrombosis, and can mediate tissue damage. In addition, NETosis is also a type of PCD that can occur in autoimmune diseases and has significant therapeutic potential.

Lysosome-dependent cell death (LDCD) is a form of PCD mediated by lysosomal contents (including proteolytic enzymes of the cathepsin family) or iron released after enhanced lysosomal membrane permeabilization (LMP) [89, 90]. It is characterized by lysosomal disruption [91] (Fig. 2M). The accumulation of intracellular ROS or the accumulation of lipid oxides can lead to lysosomal rupture, and proteolytic enzymes in lysosomes are released into the cytoplasm, leading to the occurrence of LDCD. Cathepsin is the main executor of LDCD, and blocking the expression or activity of cathepsin can reduce the occurrence of LDCD (Fig. 3M). Lysosome-dependent cell death is associated with a variety of pathophysiological conditions, including inflammation, tissue remodeling, aging, neurodegeneration, cardiovascular disease, and intracellular pathogen response.

Alkaliptosis, a Ph-dependent novel form of PCD driven by intracellular alkalization, has recently been identified as a new strategy for the treatment of a variety of tumors, especially pancreatic cancer [92, 93]. Studies have shown that intracellular alkalization can lead to JTC801-induced alkalosis, while oxidative stress is not necessary; therefore, alkaliptosis is a form of intracellular alkali-dependent regulatory necrosis with necrosis-like morphological characteristics [93] (Fig. 2N). At the molecular level, alkaliptosis may be mediated by inhibitor of nuclear factor kappa B kinase subunit β (IKBKB), which can induce the downregulation of nuclear factor κB pathway-dependent carbonic anhydrase IX (CA9), leading to alkaliptosis (Fig. 3N). Therefore, IKBKB can mediate the occurrence of alkaliptosis, while CA9 can prevent the occurrence of alkaliptosis; however, the exact mechanism involved remains unclear.

Oxeiptosis is a novel form of noninflammatory PCD induced by oxygen-free radicals and independent of caspases [94]. Oxeiptosis is morphologically characterized by apoptosis-like cell death (Fig. 2O). This process involves the interaction of a cellular ROS sensor, the antioxidant factor KEAP1, the phosphatase PGAM5, and the proapoptotic factor AIFM1 [94]. Hyperactivated KEAP1 mediates H_2_O_2_-induced oxeiptosis in an NFE2L2-independent manner by dephosphorylating AIFM1 at Ser116 through an interaction pathway involving Keap1-PGAM5 (Fig. 3O). Oxeiptosis is important for preventing inflammation caused by ROS or ROS-producing agents such as viral pathogens.

## Concluding remarks

In recent years, with the in-depth study of PCD, several new cell death pathways have been identified. Among the PCDs, apoptosis mainly shows unique morphological characteristics, such as membrane integrity, cell shrinkage, and apoptotic body formation, and does not cause an inflammatory response, while necroptosis, pyroptosis, ferroptosis, and some niche cell death forms, such as NETosis, mainly show necrosis-like morphological characteristics, such as cell swelling, cell membrane rupture, organelle collapse, and an inflammatory response. In addition, it is worth noting that some of the forms of niche cell death mentioned above may not be a separate type of cell death or an independent signaling pathway but rather a side effect or phenomenon that occurs in typical cell death processes, such as apoptosis, necroptosis, pyroptosis and ferroptosis. Growing evidence highlights the “communication” between different cell death pathways [95].

Multiple modes of PCD often occur, and loss of control of single or mixed types of cell death leads to human diseases, such as cancer, hematologic disorders, autoimmune deficiency disorders, neurodegeneration, and infectious diseases. Moreover, PCD not only plays a regulatory role in maintaining the homeostasis of the body but also may play a regulatory role in unnecessary cell death. An increasing number of researchers have studied the cell death pathway as a drug target. Therefore, it is very important to clarify the mechanism and biomarkers of cell death for research on clinical drugs, especially antitumour drugs (Table 2).

**Table 2:** Biomarkers of various PCDs and their inducers and inhibitors.

PCD pathway	Biomarkers	Inducers	Inhibitors (target)
Apoptosis	Casp3,8,9 BCL-XL BCL-2 Fas	TNF + Smac-mimetic TNF + ActD TNF + CHX Trail + Smac-mimetic Fas Ligand + Smac-mimetic	Z-VAD-fmk (Pan Casp) Z-IETD-fmk (Casp8) Z-DEVD-fmk (Casp3)
Necroptosis	RIPK1 RIPK3 MLKL	TNF + Smac-mimetic + Z-VAD-fmk Trail + ActD + Z-VAD-fmk Fas Ligand + CHX + Z-VAD-fmk	Necrostatin-1 (RIPK1) Necrostatin-1s (RIPK1) GSK872 (RIPK3) Necrosulfonamide (MLKL)
Pyroptosis	Casp1,11 GSDMD IL-1β,18 NLRP3	LPS + Nigericin	Ac-YVAD-cmk (Casp1) Wedelolactone (Casp11) Ac-FLTD-CMK (GSDMD) MCC950(NLRP3) Disulfiram(ALDH1)
Ferroptosis	Fe^2+^ GSH GPX4 ROS	Erastin Glutamate Buthionine Sulfoximine (1 S,3 R)-RSL3 FIN56	Deferoxamine (Fe^2+^) Deferiprone (Fe^2+^) Cyclipirox (Fe^2+^) Ferrostatin-1(ROS) Selenium (GPX4)
Autophagy-dependent cell death	Atg ClassIII PI3K Beclin1 LC3	Tat-Beclin1 BredeldinA C2-ceramide Rapamycin	Chloroquine (Lysosome) Concanamycin A (H^+^-ATPase) 3-methyladenine (ClassIII PI3K)
Mitotic Catastrophe	Casp3 P53 CyclinB1/CDK1	UV Cisplatin Microtubule targeting agents Adriamycin Radiation	NA (Not Applicable)
Immunogenic cell death	CRT ATP HMGB1 1-IFN ANXA1	Viral infection Anthracyclines Bortezomib Adriamycin Hypericin-based photodynamic therapy Radiation	ENTPD1 (ATP) NT5E (ATP)
Entosis	RhoA ROCK1,2 CDH1	CDH1	C3-toxin (RhoA) Y-27632 (ROCK) Blebbistatin (Myosin)
Parthanatos	AIFM1 MIF PARP-1	UV IR Alkylating agents Oxidative stress	BYK204165 (PARP-1) AG-14361 (PARP-1) Iniparib (PARP-1)
Cuproptosis	Cu^2+^ FDX1 DLAT LIAS	Elesclomol + Cu^2+^ NSC-319726 + Cu^2+^ Disulfiram + Cu^2+^	Tetrathiomolybdate (Cu^2+^)
Disulfidptosis	SLC7A11 NADPH Cystine Disulfide	KL-11743 Bay-876	Dithiothreitol (Disulfide) β-mercaptoethanol (Disulfide) TCEP (Disulfide)
NETosis	NETs NADPH MPO ROS	Phorbol myristate acetate IL-8 LPS	Tetrahydroisoquinolines (NETs) Cl-amidine (PAD4)
Lysosome-dependent cell death	LMP Cathepsins STAT3 ROS Fe^2+^	Lysosomotropic detergents Dipeptide methyl esters Sphingosine Phosphatidic acid	Leupeptin hemisulfate(Cathepsins) Deferoxamine (Fe^2+^) NAC (ROS) CA-074Me (Cathepsin B)
Alkaliptosis	IKBKB CA9	JTC801	IMD0354 (IKBKB) CAY10657 ((IKBKB) N-acetyl alanine acid (pH)
Oxeiptosis	ROS PGAM5 KEAP1 AIFM1	Ozone H_2_O_2_	NAC (ROS)

There is a lack of a more rational classification of the cell death pathways that have been identified thus far. In addition, the specific mechanism of some cell death signaling pathways is not very clear, and the relationship between different types of cell death is poorly understood. For some pathways, there are still great controversies in the academic community, and further extensive and in-depth research is needed. The regulatory mechanisms of PCD on living organisms from micro- to macrosystems are the keys to the continuation of life, and a deep understanding of these mechanisms is extremely important for human development.
